# SPEED: an integrated, smartphone-operated, handheld digital PCR Device for point-of-care testing

**DOI:** 10.1038/s41378-024-00689-2

**Published:** 2024-05-20

**Authors:** Haoqing Zhang, Xiaocheng Liu, Xinlu Wang, Zhiqiang Yan, Ying Xu, Martina Gaňová, Tomáš Řezníček, Marie Korabečná, Pavel Neuzil

**Affiliations:** 1 Ministry of Education Key Laboratory of Micro and Nano Systems for Aerospace; School of Mechanical Engineering, Northwestern Polytechnical University, 127 West Youyi Road, Xi’an, Shaanxi 710072 PR China; 2 The Key Laboratory of Biomedical Information Engineering of the Ministry of Education; School of Life Science and Technology, Xi’an Jiaotong University, Xi’an, Shaanxi 710049 PR China; 3 Bioinspired Engineering and Biomechanics Center (BEBC), Xi’an Jiaotong University, Xi’an, 710049 PR China; 4 School of Marine Science and Technology, Northwestern Polytechnical University, Xi’an, Shaanxi 710072 PR China; 5 Central European Institute of Technology, Brno University of Technology, Purkyňova 123, 61300 Brno, Czech Republic; 6 ITD Tech S.R.O, Osvoboditelu, 1005, 735 81 Bohumín, Czech Republic; 7 Institute of Biology and Medical Genetics; First Faculty of Medicine, Charles University and General University Hospital of Prague, Albertov 4, 12800 Prague, Czech Republic

**Keywords:** Microfluidics, Electrical and electronic engineering

## Abstract

This study elaborates on the design, fabrication, and data analysis details of SPEED, a recently proposed smartphone-based digital polymerase chain reaction (dPCR) device. The dPCR chips incorporate partition diameters ranging from 50 μm to 5 μm, and these partitions are organized into six distinct blocks to facilitate image processing. Due to the superior thermal conductivity of Si and its potential for mass production, the dPCR chips were fabricated on a Si substrate. A temperature control system based on a high-power density Peltier element and a preheating/cooling PCR protocol user interface shortening the thermal cycle time. The optical design employs four 470 nm light-emitting diodes as light sources, with filters and mirrors effectively managing the light emitted during PCR. An algorithm is utilized for image processing and illumination nonuniformity correction including conversion to a monochromatic format, partition identification, skew correction, and the generation of an image correction mask. We validated the device using a range of deoxyribonucleic acid targets, demonstrating its potential applicability across multiple fields. Therefore, we provide guidance and verification of the design and testing of the recently proposed SPEED device.

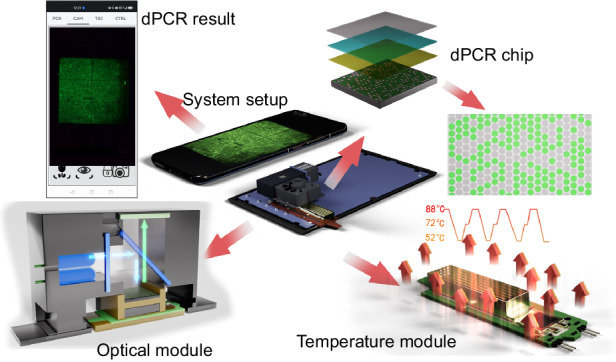

## Introduction

Polymerase chain reaction (PCR), is one of the most consequential scientific innovations of the last century, which has revolutionized the fields of genetics, diagnostics, and forensic science^1,2^. The capabilities of PCR stem from its ability to selectively amplify target deoxyribonucleic acid (DNA) sequences^1^. A third generation of PCR techniques was recently developed within this groundbreaking environment: digital PCR (dPCR). Its development, which emerged two decades ago, was primarily driven by advancements in microfluidic technology^3^.

dPCR differs from other PCR systems by compartmentalizing the PCR master mix into thousands or even millions of partitions, either in wells^4^ or emulsion droplets^5^ called either chip-based dPCR (cdPCR) or droplet-based dPCR (ddPCR). This compartmentalization often results in having one or no target molecules in each partition, effectively digitizing the distribution of DNA targets^3^. This distribution enables the application of Poisson statistics to evaluate the original copy numbers (*cn*) of the target DNA. This method bypasses the limitations of standard PCR and qPCR, offering absolute quantification of *cn* without requiring a standard PCR curve^3^. Furthermore, it aids in detecting rare targets among more abundant DNA fragments, as well as the ratio of two or more targets, providing invaluable information in the fields of medical and biological sciences^6^.

Currently, commercial dPCR devices based either on cdPCR^7^ or ddPCR^8^ typically consist of three main components: a sample loading system, a thermal cycling system, and an image processing or flow cytometry system. However, the separation of these systems from each other results in devices that are bulky, labor-intensive to operate, and expensive, with compatible consumables also being quite costly. These factors create significant barriers to the adoption of commercial dPCR devices in point-of-care testing (POCT), where there is a demand for affordable, user-friendly, and portable solutions. Numerous efforts have been made to miniaturize dPCR devices in response to this challenge with a primary focus on enhancing the structure of the dPCR chip^9,10^. These newly developed compact systems often employ thermoelectric element (TEC) also known as Peltier element, for heating and cooling^9,11^ and have emerged as viable alternatives to traditional commercial devices. The integration of smartphone-based optical detection and remote control systems further contributes to the miniaturization of dPCR devices, making them more compact, affordable, and robust. This advancement has led to promising results in bioanalytical and diagnostic applications^12^. Several smartphone-based dPCR devices have already been developed and are being used in these applications.

One representative prototype was developed with a size of ≈ (90 × 90 × 100) mm^3^ and a weight of ≈ 0.5 kg^13^. This system utilized a polydimethylsiloxane (PDMS)-based self-priming dPCR chip with 4096 partitions and a TEC-based thermal system, achieving heating and cooling rates of ≈ 5 K·s^−1^ and ≈ −4 K·s^−1^, respectively. The fluorescence image of the dPCR chip was captured and then analyzed by a smartphone using the developed application (app). Additionally, the APP controlled the PCR protocol settings and temperature. Another portable, smartphone-based cdPCR device characterized by its compact size and lightweight nature was proposed^4^. This device utilized a PDMS-based dPCR chip with 8100 partitions and was heated via the plasmonic effect and cooled with a fan, achieving maximum heating and cooling rates of ≈ 10.7 K·s^−1^ and ≈ −8 K·s^−1^, respectively. Images of the chip were captured using a fluorescence microscope, and the results were analyzed with a Python program-based algorithm. However, the PDMS dPCR chip-based device requires an external bulky instrument, such as a vacuum pump, for sample loading. Moreover, the PDMS is porous; thus, a multilayer dPCR design is required to eliminate sample evaporation during thermal cycling. Additionally, the PDMS-based dPCR chip was only used once, thus increasing the cost of a single test. Building on these advancements, we recently developed a fully integrated, smartphone-operated, handheld Si-based dPCR device—SPEED—that measures only ≈ (100 × 200 × 35) mm^3^ in size and ≈ 400 g in weight. The Si-based dPCR chip is free of bulky instruments for sample loading and allows for repeated utilization, thereby significantly lowering the running cost of the system. Designed for point-of-care-testing (POCT) applications, this device employs miniaturized thermal cycling and optical modules, completing a three-step PCR protocol in ≈ 49 min, and captures fluorescence images of the chip post-PCR^14^.

**Fig. 1:**
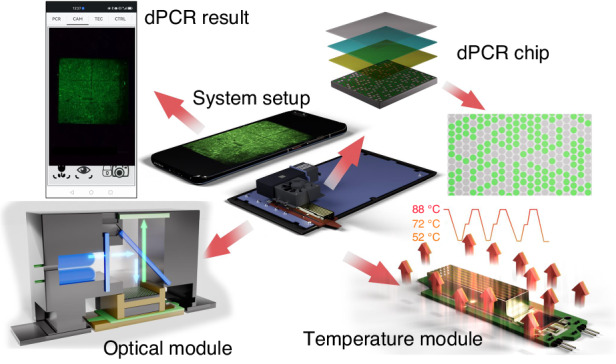
Schematic of the entire device, along with the description of discrete systems. The system is integrated into an autonomous unit with a size of (100 × 200 × 35) mm^3^. It includes a Huawei P40 smartphone and is powered by a 12 V direct current (DC) power supply. The hardware consists of a fluorescence imaging system using a light emitting diode (LED) for illumination and temperature control to control the temperature of the Si chip. The internal app in a smartphone can manually control the PCR protocol and the TEC, and the chip fluorescence image can be captured once the PCR is completed. The data are processed externally, and suitable communication software downloads the results to the smartphone screen

This study elaborates on the SPEED device development, including the design and fabrication of dPCR chips and coverslips, the optimization of temperature control design and ramping rates, the creation of an optical housing design, correction for light nonuniformity, and the development of an image processing algorithm (Fig. 1). Building on our previous work, we propose that the specifics shared herein will prove instrumental for researchers aiming to develop POCT-based devices. We therefore designed and tested a protocol for POCT-based device development and verification.

## dPCR chip design and fabrication

### dPCR theory and chip design

**Fig. 2:**
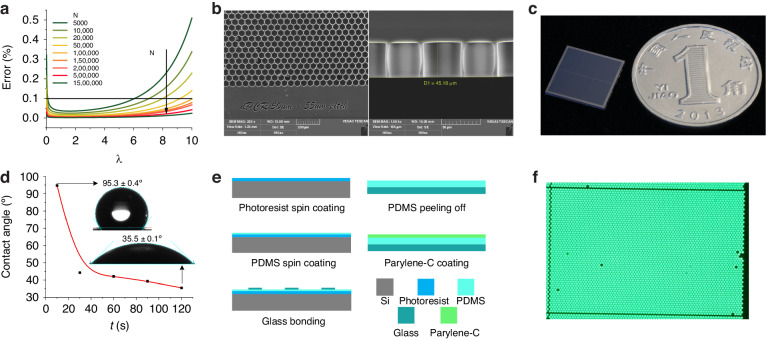
The characterization of dPCR chip. **a** The relative error as a function of an average number of copies with different partitions of the dPCR chip; (**b**) the design of the dPCR chip; (**c**) the image of the Si-based dPCR chip; (**d**) the Si surface contact angle as a function of O_2_ plasma treatment time; (**e**) the fabrication process of PDMS-Parylene-C-coated cover glass: the photoresist was spin-coated on a Si wafer as a sacrificial layer; then, a layer of PDMS was spin-coated on top of the photoresist; the cover glass was bonded to PDMS after oxygen plasma treatment; the PDMS glass was peeled off from the photoresist using acetone; finally, a layer of Parylene-C was coated on top of PDMS; (**f**) the image of one block of the dPCR chip filled with fluorescein after thermal cycling

In dPCR, each partition typically contains either no copy or a single copy of the target DNA. A target’s original *cn* is determined by counting the number of partitions, including the target, which is also known as a positive partition (*PP*). However, there are instances in which more than one copy of the target DNA is distributed into a single partition. The Poisson distribution is applied when calculating the dPCR results^15^ to accommodate this occurrence. The relative error of these results depends on the total number of partitions (*N*):

1 $${Error}=\max \left(\left|1-{e}^{\pm 1.96\frac{\sqrt{{e}^{{\rm{\lambda }}}-1}}{{\rm{\lambda }}\sqrt{N}}}\right|\right)$$

where λ is the average *cn* value of the target in a single partition. The relative error of the detection results improved markedly with increasing *N* (Fig. 2a). In addition, partition uncertainty also impacts the relative error. Thus, we use microwells instead of droplets for our dPCR chip partitions due to their lower partition uncertainty^16,17^. We also designed a series of chips with partition diameters of 50 μm, 20 μm, 10 μm, and 5 μm, resulting in total partition counts of 26,448, 139,986, 475,272, and 1,656,000, respectively (Fig. 2b). This design flexibility, which offers varying numbers of partitions, enables us to meet diverse dPCR sensitivity requirements. Additionally, the chip size was designed to be (9 × 9) mm^2^ to avoid exceeding the high-power TEC dimensions of ≈ (14 × 14) mm^2^ used in our SPEED system (Fig. 2b). We divided all partitions into six distinct blocks to simplify identification and subsequent image processing. Thus, all chip types are compatible with the image processing algorithm^18–20^. However, the increasing number of partitions complicates the chip design and fabrication because the partition positions need to be properly organized to improve sample loading and avoid sample evaporation. In addition, increasing the number of partitions per image compromises the image quality per partition and challenges image processing because the number of pixels per partition becomes insufficient, and image stitching should be considered.

### dPCR chip fabrication and surface treatment

We selected Si as our dPCR chip substrate due to its superior thermal conductivity and ease of mass production, both of which contribute to faster detection and lower costs. The chip fabrication process began with an ≈ 100 mm Si wafer spin-coated with an ≈ 3.8 μm thick positive photoresist at 1800 RPM. This wafer then underwent a prebake at ≈ 110 °C for ≈ 80 s and was subsequently exposed to ultraviolet light for ≈ 10.5 s through a patterned Cr layer on a soda lime mask at an energy of ≈ 9.3 mJ·cm^−2^. The exposed wafer was postbaked at ≈ 120 °C for ≈ 180 s, and the pattern was then developed using a tetramethyl ammonium hydroxide-based developing solution for ≈ 27 s, followed by rinsing with deionized water (DI H_2_O) and drying with N_2_ gas. The next step involved deep reaction ion etching to achieve depths and well diameters of ≈ 30 μm, ≈ 20 μm, ≈ 10 μm, and ≈ 5 μm. The finishing touch involved removing the residual photoresist using O_2_ plasma, dividing it into 54 individual chips using a diamond blade dicing saw, and removing the photoresist protective layer with acetone followed by propane-2-ol (known as isopropanol or IPA), a DI H_2_O rinse, and N_2_ blow drying (Fig. 2c). Details of the dPCR chip fabrication were described earlier^19^.

We treated the chip surface with O_2_ plasma for 120 s to reduce the contact angle from 95.3° to 35.5°, thus making the surface hydrophilic (Fig. 2d). This treatment enabled easier loading of the PCR master mix into the partitions. In parallel, we fabricated a protective cover using a ≈ (12 × 12) mm^2^ microscope coverslip coated with ≈ 50 µm of polydimethylsiloxane (PDMS) and ≈ 2 µm of Parylene-C. The PDMS was first spin-coated at ≈ 1500 RPM for ≈ 30 s and cured on a ≈ 100 mm Si wafer covered with a photoresist as a sacrificial layer. Then, we bonded it to the cover glass and detached the PDMS-coated coverslip from the wafer by dissolving the photoresist layer in acetone and then depositing a Parylene-C layer atop the PDMS (Fig. 2e).

We validated the performance of the coverslip by pipetting ≈ 3 µL of an ≈ 1.5 mM fluorescein solution to the edge of the chip and spreading it to fill all partitions using the coverslip. We then added ≈ 10 µL of mineral oil to the edge of the coverslip to guard against evaporation and cross-contamination of fluorescein in the microparticles. The dPCR chip was positioned on a TEC and placed beneath an objective camera lens to initiate the PCR protocol. During the manipulation, it was essential not to move or touch the glass surface; otherwise, clusters and bubbles between fluorescein and oil were created at high temperatures. We obtained images of the same block of the chip before and after performing the 40-cycle PCR protocol. There was little fluorescein evaporation in a few wells, verifying the performance of the proposed PDMS and the Parylene-C-based cover glass (Fig. 2f).

## Temperature control system

### Hardware

The temperature cycling of the dPCR chip was enabled by a high-power density TEC, with an exceptionally high maximum dissipated power density of ≈ 17 W·cm^−2^. The TEC was soldered onto a copper system, thereby efficiently managing heat dissipation to prevent system overheating. We affixed an Au-plated brass holder on top of the TEC to accommodate the dPCR chip. Additionally, the surfaces of the TECs were coated with In/Sn alloy, a substance with a melting temperature of approximately ≈ 120 °C. This melting temperature of the coating ensured that our soldering process did not damage the internal components of the TEC, as the materials were soldered with a melting temperature of ≈ 180 °C.

**Fig. 3:**
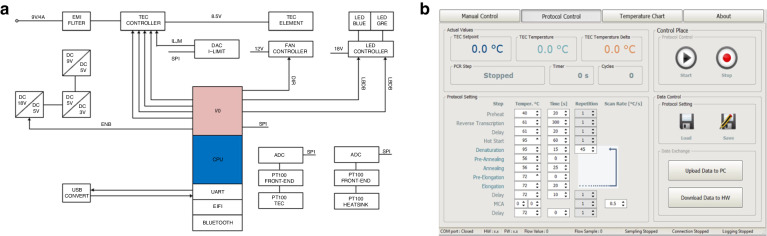
Hardware and software design of the device. **a** Schematic diagram of the SPEED hardware and (**b**) user interface of the PC

The TEC was driven by an H-bridge, fed with electrical current pulses operating at a nominal frequency of ≈ 100 kHz and a maximum amplitude of ≈ 5 A. A schematic diagram of the entire system is shown in Fig. 3a. These pulses were filtered by an inductor/capacitor circuit, resulting in effective powering of the TEC with a DC signal, thus minimizing the system noise generated during the heating process. Control over the TEC’s temperature transition (under heating or cooling) was managed by the central processing unit. This control was achieved through the regulation of the electrical current duration and direction using the pulse width modulation method. A cavity was fabricated within the brass holder next to the dPCR chip to accommodate a resistive temperature detector (RTD) of the Pt100 type.

We implemented a proportional integrative derivative (PID) closed feedback loop system to maintain accurate control over the temperature of the top TEC plate and to follow the desired PCR protocol. The operational effectiveness of this system was based on the signals received from the Pt100 type of RTD.

### Software

The implemented software consists of three distinct packages: firmware within the system’s central processing unit (CPU); PC/Android-based software for user interfacing, as shown in Fig. 3b; and remote software for data analysis and processing. The system can be controlled either from a smartphone or via a PC, considering that PC control may be more convenient (e.g., for protocol development).

#### Firmware

The firmware, which is written in C code, was loaded directly into the system CPU’s memory. It managed multiple tasks, including controlling the temperature of the dPCR chip, directing power dissipation in the TEC, adjusting the direction of electrical current for heating or cooling the top TEC plate, controlling the fan’s rotation, and adjusting the LED illumination power. This comprehensive protocol allowed for two- or three-step PCR or RT‒PCR protocols, including speed-up versions, where annealing and elongation could optionally consist of two steps.

#### PC-based Software

Our Windows-based software is compatible with all PCs utilizing 32×/64× architectures, offering complete control over all dPCR hardware functions through a USB interface. This control includes the ability to upload PCR protocol parameters and PID constants for the TEC controller. The software features a user-friendly graphical user interface (GUI) that enables easy manipulation of the dPCR system. It provides two modes: manual mode for testing individual functions and protocol mode for automatic control according to specific PCR protocol settings. Additionally, the software logs real-time temperature data, thereby delivering detailed insights into hardware status at any given moment. Temperature data from both the heater and the TEC cooler can be downloaded onto the PC in American standard code for information interchange (ASCII) format for further analysis, thus aiding in system performance improvement.

#### Android-based APP for Control and IoT

**Fig. 4:**
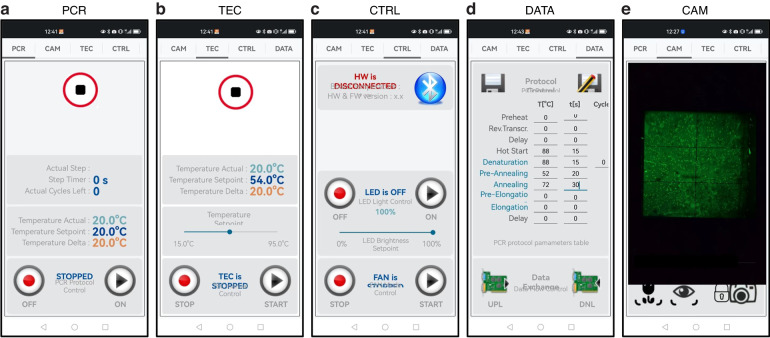
The user interface of the Android-based app included. **a** the PCR status, (**b**) the TEC status, (**c**) the control page, (**d**) the dPCR protocol, and (**e**) the camera module

Our Android-based control software, which operates on the attached P40 smartphone (Huawei, Inc.), features a GUI for intuitive use and manages all primary dPCR hardware functions via a Bluetooth communication protocol. The software includes four distinct modes: the PCR window for monitoring both set and actual TEC temperatures (Fig. 4a), including the timing of each PCR step; and the TEC window for manually controlling the TEC within its operational range (Fig. 4b); the CTRL window for testing the cooling fan and LED intensity (Fig. 4c); the DATA window for setting the PCR protocol (Fig. 4d). Additionally, there is a CAM mode for controlling the camera, and capturing dPCR images (Fig. 4e), processing data via remote computer control, and displaying the results. Captured images are transmitted over 4 G/5 G networks to an external computer, which then returns the processed results for display.

Rapid advancements in Internet of Things (IoT) technology and big data analytics provide opportunities for POCT to serve as alternatives to traditional, labor-intensive, and time-consuming data analysis techniques^21,22^. IoT-assisted systems, including microfluidic platforms and data-processing algorithms, have shown significant potential for POCT. For example, the IoT PCR system and other microfluidic systems for disease diagnosis have been successfully implemented^23,24^. Here, we have demonstrated the use of a smartphone platform for dPCR system programming and image processing, indicating potential integration with IoT systems for disease monitoring. Data collected from portable devices are transmitted from smartphones or local computers to a cloud server, facilitating real-time data analysis. This process makes detection data more accessible and easy to display on a smartphone screen. The development of an application for smartphones that leverages cloud computation simplifies the system by enabling data storage, result sharing, and data transfer to a central hub for efficient health monitoring and treatment^25^.

### Temperature profile and uniformity

**Fig. 5:**
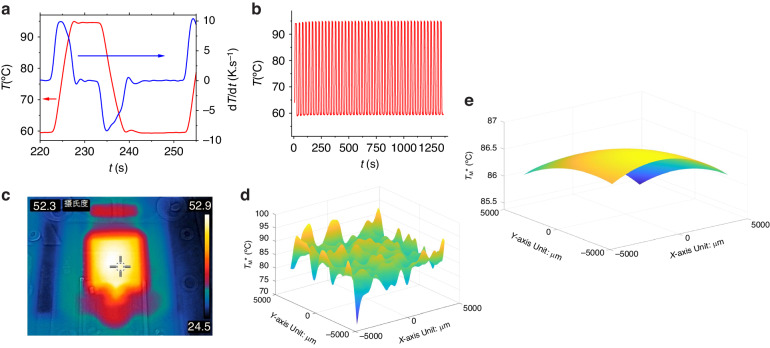
Temperature cycling profile. **a** The dPCR temperature protocol for a single 2-step cycle is shown, depicting the temperature profile in red and the heating/cooling rate in blue. **b** The dPCR temperature protocol for 45 cycles is illustrated, highlighting the temperature stability between cycles and throughout the entire PCR time. **c** An infrared image of the dPCR chip holder set to 52 °C. **d** The MCA-based temperature uniformity determination is presented. **e** The temperature uniformity extracted from the MCA-based measurements is shown and used for improvements in temperature mapping

We assessed the heating and cooling rates of our system by recording a single PCR cycle (Fig. 5a red line) within a two-step thermal protocol: ≈ 10 s of denaturation at ≈ 95 °C and ≈ 20 s of annealing at ≈ 60 °C. The combination of closed feedback loop control with a high power density TEC leads to heating and cooling rates of ≈ 9.9 °C·s^−1^ and ≈ −8.5 °C·s^−1^, respectively (Fig. 5a blue line). We then executed 45 cycles of the entire two-step dPCR protocol (Fig. 5b), which were completed in ≈ 25 min.

The performance of a heating/cooling system typically follows a first-order exponential function that is derived from the heat balance equation. In this function, the temperature recorded at the sensor (*T*_s_) lags behind the heating/cooling power due to a delay in dissipated power (*P*_d_). The response of the system is governed by the following equation:

2 $${T}_{{\rm{s}}}={T}_{1}\left(1-{\triangle T\cdot e}^{-\frac{t}{{\rm{\tau }}}}\right)$$

In Eq. (2) ∆*T* represents the temperature change at a steady state resulting from *P*_d_, expressed as ∆*T* = *P*_d_/*G*, where *G* is the thermal conductivity of the system. In this context, τ is then *H*/*G* with *H* representing the heat capacitance of the thermal system.

The operation of this control system is well understood and generally proceeds smoothly after suitable PID constants of the feedback loop system are set. Nevertheless, challenges may emerge due to the finite heat transfer rate between the heater and the sample. This leads to a delay in the temperature profile of the sample, particularly during transition periods, compared to the temperature profile of the heater. The temperature of the PCR mixture, not the temperature of the heater, is crucial for accurate reaction kinetics. Therefore, we investigated the delay between the temperature of the TEC and the sample and the heat transfer rate by conducting the protocol using a dPCR chip filled with fluorescein as a temperature sensor^26^. The software for PCR protocol control was developed to facilitate this technique by enabling the addition of preannealing and preelongation steps, thereby accelerating the cooling and heating of the sample.

This additional step deliberately overshoots the heater temperature, thereby accelerating the cooling and heating rates of the master mix. We referred to these steps as preannealing and preelongation. The duration of these steps was determined based on the heat transfer between the heater and the sample. The timing of the protocol was established such that the total duration of the elongation and annealing steps remained constant, even after the inclusion of the preelongation or preannealing steps. As a result, the heating and cooling of the sample accelerated.

Temperature uniformity across the chip is paramount, as it directly impacts PCR efficiency and influences the uniformity of *PP*s and the precision of the results. Typically, thermal uniformity is gauged using a mid-range infrared (IR) camera that operates in a window from ≈ 8 to ≈ 14 µm. However, the IR system measures the power of the radiation emitted (*P*_R_) from the sample surface instead of the actual temperature, as prescribed by the Stefan–Boltzmann law^27^. Considering that the glass used in our system is opaque in the IR range, this method only provides information about the surface temperature rather than the temperature of the PCR master mix, which needs monitoring. Nevertheless, we captured the IR image of the chip heater at ≈ 52 °C (Fig. 5c), but the data showed insufficient temperature resolution.

We thus measured the temperature uniformity of the sample within the dPCR chip by employing a DNA melting curve analysis (MCA)-based technique. This method has previously been utilized for PCR samples^28^ and microcalorimeters^29^, allowing us to ascertain the corresponding heat transfer rates^30^ and temperature nonuniformity^31^ of the dPCR chip. We filled the chip with a PCR master mix containing a dsDNA template with a known melting temperature (*T*_M_) of ≈ 87 °C, as verified by a commercial qPCR system. A series of fluorescence images were captured at various temperatures, and an MCA was performed on all partitions. We obtained an apparent melting temperature (*T*_M_*) of (86.41 ± 0.26) °C (mean value ± σ from 26,448 partitions) (Fig. 5d). The difference between the *T*_M_* mean value and the PCR master mix *T*_M_ is used for temperature sensor calibration, with the σ value serving to determine the nonuniformity of the dPCR chip temperature. We also created a 2D map of the chip using the *T*_M_* value as a visualization parameter (Fig. 5e). This map could highlight any uniformity issues related to heat transfer between the chip and the TEC.

## Optical system

### Optical design

Our optical design incorporated the use of four ≈ 470 nm LEDs as our primary light sources, powered by a maximum current set to 40 mA. Based on these attributes, the design did not necessitate the inclusion of a focusing lens. The light produced by the LEDs was subsequently channeled through a bandpass filter, with a nominal center wavelength of ≈ 470 nm and a bandwidth of 40 nm. The filtered light was then redirected at a ≈ 90° angle by a dichroic mirror with a cutoff wavelength of 500 nm and guided onto the dPCR chip. The light emitted from the PCR master mix within the partitions was bounced back through the dichroic mirror. Any remaining blue light was then filtered out by an excitation bandpass filter with a center nominal wavelength of ≈ 525 nm and a bandwidth of ≈ 50 nm. The light filtered through this process was captured by a camera integrated into the Huawei P40 smartphone. Camera performance affects the system, and cameras on different phones have different resolutions, minimal focusing distances, sensitivities, and especially different phone locations; thus, the system must be designed for specific cell phones. We designed our system for smartphone operation as typical smartphones have sophisticated cameras with macro functions for facilitating the close-up photography that is required. However, mobile phone-based cameras cannot compete with fluorescence microscopy because the cost of fluorescence microscopy with decent optics is 50 to 100 times greater than that of mobile phones providing images of higher quality than the images from smartphones. Even so, a Huawei P40 provides dPCR chip images of satisfactory quality for their further processing.

**Fig. 6:**
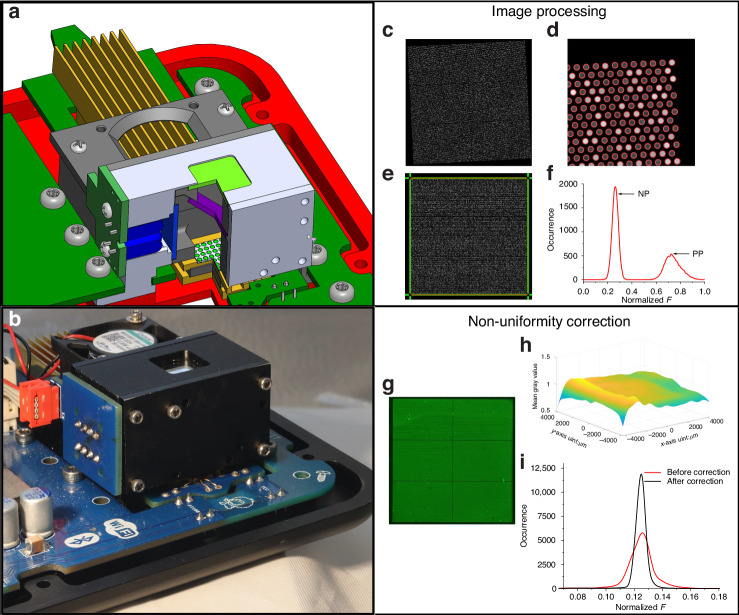
The schematic of the optical design and image processing algorithm. The optical system, which includes (**a**) the optical design showing a cross-section of the fluorescent system and (**b**) its optical image, image processing schematic, and image nonuniformity correction schematic. In the image processing schematic, (**c**) the emulated image of the dPCR chip was generated, and (**d**) the partitions were recognized by the circular Hough transform; (**e**) then, FFT and 3D projection were implemented to rotate the image, and relocate the partitions, respectively; and (**f**) the partition number as a function of the extracted F values of each partition was finally constructed. The schematic of nonuniformity correction: (**g**) the fluorescence image of the dPCR chip filled with master mix before correction; (**h**) the generated 3D fluorescent intensity distribution map from (**f**) and (**i**) the comparison of histograms from images before and after correction

We engineered an optical housing from an aluminum alloy to optimize the quality of the fluorescence images further. This construction process was executed using computer numerical control machining, followed by the deposition of a layer of camphor^24^. As a result of the campor application, we achieved a surface reflection reduction of approximately 96%, which significantly enhanced the quality of the fluorescence images (Fig. 6a, b).

### Image processing and illumination nonuniformity correction

Using a recently developed dPCR emulation program, we generated a fluorescent dPCR image to illustrate the structure of our image processing algorithm^20,32^. Monochromatic images are easier to process than colored images, so we first converted the captured fluorescence image into a monochromatic format (Fig. 6c). We then identified the organized circular partitions in the chip using the circle Hough transform algorithm (Fig. 6d). Subsequently, we introduced a fast Fourier transform-based skew correction algorithm to rectify the rotated image. Additionally, we developed and implemented a 3D projection transformation to accurately identify all partitions’ coordinates (Fig. 6e). The specific details of these algorithms can be found in our previous publication^20^.

We then proceeded to extract the fluorescence amplitude (*F*) from each partition, creating an occurrence histogram as a function of the *F* values (Fig. 6f). However, the problems of nonuniform chip illumination and nonuniform fluorescence imaging often result in fluorescence image inhomogeneity. To address this issue, we filled the PCR chip with ≈ 0.15 µM fluorescein, yielding *F* values similar to those in the PCR master mix post-PCR with EvaGreen or FAM probes. We then captured images of the chip at room temperature (Fig. 6g). We extracted the *F* values using the previously mentioned algorithm and employed a Gauss filtering algorithm to generate a map that depicts the distribution of the *F* values. This map displayed inhomogeneity in the fluorescence image caused by nonuniform illumination, inconsistent fluorescent signals, and camera-induced optical effects (Fig. 6h). We subsequently used it to create an image-correction mask to correct the *F* values of the original fluorescence images by dividing them by the corresponding *F* values from the mask. This method significantly reduced the half-width of the resultant histogram after correction, dropping to 0.61 from 1.609 before correction (Fig. 6i).

## Data analysis

We subjected our SPEED device to rigorous validation tests using a range of synthesized DNA targets associated with pancreatic tumorigenesis, SARS-CoV-2, and chromosomes 21 (Chr21) and 18 (Chr18). This process enabled us to effectively detect genetic changes typical for pancreatic cancer, COVID-19, and Trisomy 21 syndrome. Additionally, the versatility of the device is evident through our demonstration of its duplexing capacity. We amplified the viral genes N and E for SARS-CoV-2 detection and conducted a concurrent analysis of sequences from Chr21 and Chr18 for use in non-invasive prenatal diagnostics. Our previously published paper offers a detailed account of the underlying dPCR protocols and results^14,33^.

**Fig. 7:**
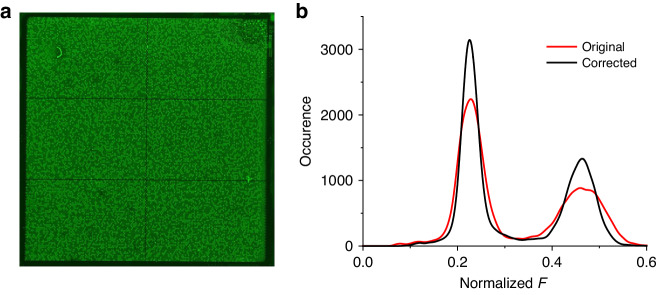
dPCR results. **a** Original dPCR image; the image after the nonuniformity correction is not shown here. **b** The occurrence as a function of F values extracted from images before and after the correction illustrates the image improvement

We further conducted a dPCR experiment using a synthesized DNA target related to pancreatic tumorigenesis to elucidate our data analysis process. We employed a *cn* of 18,514 in the dPCR chip, resulting in an average *cn* per partition (λ) of 0.7. The PCR master mix and the dPCR protocol we used were consistent with our earlier study^14^. Upon completing the dPCR, we captured a fluorescence image of the dPCR chip (Fig. 7a). This image was corrected using a previously generated mask and processed in a manner analogous to the fluorescein images. Following this procedure, we obtained *F* values for all partitions. These values were then used to construct a histogram that was fitted with a twin-peak Gaussian function. The first and second peaks of the function corresponded to negative partitions (*NP*s) and *PP*s, respectively. The total counts were calculated by dividing the respective areas under the Gaussian curves by the histogram’s bin size. We utilized Gaussian multipeak curve fitting when partitions had differing *cn* values. The resulting half-width values of the Gaussian curve improved from 5.97 ± 0.04 and 11.23 ± 0.15 to 3.97 ± 0.04 and 7.31 ± 0.12 for *NP* and *PP*, respectively.

Differentiating between multiple peaks proved to be challenging, and this process did not effectively remove the *F* value differences for partitions containing one or more DNA strands. We calculated the areas representing *NP*s and *PP*s by integrating the area and then dividing the result by a bin number of 128 to address this problem (Fig. 7b). Next, we used the Poisson distribution to convert the number of *PP*s into the original DNA *cn* value. The *cn* value obtained from the experiment was 0.61; the discrepancy between the calculated and extracted λ is likely attributable to sample evaporation at the edge partitions of the chip. In addition, we verified the device with λ values ranging from 0.6 to 0.1 and the duplexing methodology using different ratios of two genes in another paper. The expected values matched the calculated values, and we believe that the device is repeatable^14^.

## Conclusion

This study demonstrated the design, fabrication, and application details of a smartphone-powered dPCR device, denoted SPEED. The system can be controlled by a smartphone using a Bluetooth protocol or from a PC using a USB interface. The latter is convenient for protocol development, while the former allows the system to be highly compact. We demonstrate the technology’s effectiveness by outlining methodologies for chip design and fabrication, temperature control, optical system design, and comprehensive data analysis. The precision and efficiency of dPCR analysis are enhanced through the use of Si chip design, microwell partitioning, improved temperature control, an optimized optical system, and robust image processing algorithms. The validation of SPEED for use with a variety of synthesized DNA targets, including those relevant to pancreatic cancer, COVID-19, and chromosomal disorders, highlights its extensive potential in molecular diagnostics. In conclusion, this work represents a significant advancement in smartphone-powered dPCR technology, pushing the boundaries of PCR systems toward portable, affordable, and effective genetic testing solutions.
